# Effect of Peanut Butter Intake on Sleep Health in Firefighters: A Randomized Controlled Trial

**DOI:** 10.3390/ijerph21050571

**Published:** 2024-04-29

**Authors:** Tiffany J. Oberther, Andrew R. Moore, Austin A. Kohler, David H. Shuler, Nicole Peritore, Angelia M. Holland-Winkler

**Affiliations:** Department of Kinesiology, Augusta University, 3109 Wrightsboro Road, Augusta, GA 30909, USA; toberther@augusta.edu (T.J.O.); andmoore@augusta.edu (A.R.M.); akohler@augusta.edu (A.A.K.); dashuler@augusta.edu (D.H.S.); nperitore@augusta.edu (N.P.)

**Keywords:** monounsaturated fatty acids, sleep latency, sleep efficiency, sleep awakenings, peanuts, actigraphy, VAS, shift work

## Abstract

Sleep is often impaired in firefighters due to the psychologically and physiologically intense nature of their work and working shift schedules. Peanut butter is affordable and a substantial source of monounsaturated fatty acids, which may aid sleep health. Thus, this study sought to determine if a daily serving of peanut butter consumed before bedtime for seven weeks altered sleep quality and quantity among full-time firefighters. Forty firefighters (peanut butter group = 20; control group = 20) participated in this eight-week randomized controlled trial. All participants completed a subjective questionnaire on mood, focus, and alertness twice daily and wore an Actigraph wristwatch to measure sleep variables, including latency, efficiency, time in bed, time asleep, wake after sleep onset, number of awakenings, and time spent awake. After a baseline week, the peanut butter group consumed two tablespoons of peanut butter two hours prior to bedtime for seven weeks. Compared to the control group, the peanut butter group did not demonstrate significant changes (*p* > 0.05) in sleep measures or subjective feelings of mood, focus, or alertness after consuming peanut butter for seven weeks. Therefore, peanut butter as a source of peanuts did not alter sleep quality or quantity in this group of firefighters.

## 1. Introduction

Sleep is vital for cognitive functioning, physiological performance, and general health [1,2,3]. People suffering from poor sleep outcomes, such as insufficient quantity, inconsistent timing, and low efficiency, display higher rates of morbidity and mortality and are less prepared to perform physically and mentally challenging tasks [4,5]. Despite its importance, workers in some occupations are at risk of poor sleep. Firefighters typically work shifts of 24 h on/48 h off, with intermittent sleep due to calls throughout their shift which cannot be anticipated [4,5,6,7]. As a result of this type of schedule, they undergo intense occupational challenges and commonly function under circumstances of chronic sleep deficiency and circadian disruption. This sleep interruption affects cognitive performance but does not necessarily present feelings of sleepiness [8,9]. As a result, firefighters may react to an emergency and consider themselves to be satisfactorily ready but respond in a cognitively or physically impaired state [10,11]. A firefighter deprived of sleep who is physically and/or cognitively impaired represents a safety hazard to themselves, fellow first responders, and victims of fires who must be rescued. Therefore, interventions that reduce the burden of impaired sleep schedules are worthy of investigation.

Some strategies for improved sleep have been developed, such as maintaining a consistent sleep schedule and environment and limiting stressful activities prior to sleep [12]. The occupational demands and shift scheduling that firefighters face prohibit the adoption of most behavioral sleep strategies [13,14]. Nutritional interventions for improving sleep health are more feasible and may be easier to implement because food preparation and intake are somewhat flexible, and the barriers of an unknown or inconsistent work schedule can be circumvented to some degree. Diets high in protein are reported to result in fewer wake incidents [15]. Previous research has also shown that monounsaturated fatty acids (MUFAs) could promote sleep health [16,17,18,19]. Marques-Vidal et al. examined the relationship between objectively measured sleep duration and dietary intake and found that those with lower MUFA were shorter sleepers [16]. Sartotius and Haring found that MUFAs were beneficial for sleep behaviors, and in Zuraikat et al., a larger intake of legumes predicted better sleep efficiency and overall sleep quality [17,18].

Peanuts are an inexpensive, palatable, and nutritionally dense snack food that contains the nutritional elements that are beneficial to sleep. As a whole food and used as an ingredient, peanuts offer a high protein content and serve as a plentiful source of monounsaturated oil, as well as an assortment of healthy micronutrients and bioactive compounds [20,21]. The MUFA of regular peanuts in the United States (US) is 49–57% [20]. In addition to high MUFA content, peanuts also contain high levels of the micronutrients vitamin E and magnesium, which have both been associated with better sleep outcomes [1,22]. Blanched peanuts can also be processed into peanut butter with limited impact on nutrient content, increasing the portability and ease of consumption of the protein and MUFA contained in peanuts [21]. 

The dietary patterns of firefighters consist of high amounts of red meat and fast food, which leads to an overconsumption of saturated fat, cholesterol, sugar, and sodium [23,24]. Many nutritional interventions have been employed to help firefighters improve their dietary habits and overall health. While the nutrition interventions may help over the following year after the intervention, firefighters typically revert to their dietary patterns prior to the intervention in follow-up years [25]. Therefore, it is necessary to determine and promote sustainable and realistic dietary choices for firefighters to easily implement as a long-term dietary pattern. Long-term exposure to a food often leads to a decrease in palatability and reduced desire to consume the food [26,27]. However, Alper et al. demonstrated that chronic exposure to consuming peanuts did not lessen the participants’ desire to consume the peanuts, thus suggesting that peanuts may be a sustainable dietary addition [28]. Therefore, in addition to the macro- and micro-nutrient content of peanuts that may aid in sleep, long-term adherence to peanut consumption seems promising.

The potential impact of chronic peanut butter consumption on sleep health has not been examined to date. Therefore, the primary purpose of this study was to determine if a daily serving of peanut butter consumed before bedtime for seven weeks affects measures of sleep quality and quantity among full-time firefighters. The secondary purpose was to determine if subjective feelings of mood, focus, and alertness in the morning and before bedtime were altered by the regular consumption of peanut butter. We hypothesized that sleep and subjective feelings of alertness, focus, and mood will improve due to adding a realistic and palatable food source regularly prior to bedtime that contains natural sleep enhancement properties including MUFAs, vitamin E, and magnesium. 

## 2. Materials and Methods

### 2.1. Experimental Design

A parallel arm randomized controlled design was used to determine if peanut butter consumption prior to bedtime for 7 weeks alters sleep quality and/or quantity in firefighters located in the southeastern part of the United States. Baseline data were collected at each participant’s fire station and followed by a 1-week baseline period. Following the 1-week baseline period, participants were randomized by a blinded statistician to either a peanut butter (PB) group or a control group. All participants remained in their designated group for 7 weeks following the baseline week. Data were collected a second time at each participant’s fire station immediately after the 7-week intervention. In addition to the two primary data collection visits that occurred at baseline and post-intervention, data were collected daily via subjective questionnaires and actigraphy to monitor sleep. Informed consent documents were emailed to participants prior to the first baseline visit for review and then signed prior to the start of baseline data collection. This study was approved by the University’s Institutional Review Board (IRBnet ID# 1928368), and all procedures performed followed institutional guidelines. This study was registered as a clinical trial (ClinicalTrials.gov (accessed on 24 April 2024) Identifier: NCT06364202).

### 2.2. Participants

The program G*Power version 3.1 was used to determine an appropriate sample size necessary to detect a significant effect of peanut butter intake on sleep measures. The purpose of this study was novel, as there are no studies currently published that have investigated the effects of peanut butter consumption on sleep outcomes. Therefore, without a reference effect size from a comparable study, we selected a small-moderate effect size (f = 0.175), an alpha level of 0.05, and a power level of 0.80 for the power analysis. For a mixed ANOVA with two independent groups and data collection at eight timepoints, an estimated 32 participants would be required to detect a significant effect based on these parameters. Additional participants were recruited to protect against potential participant dropout and data loss. In total, 40 full-time firefighters (39 males, 1 female) were recruited from the local fire department and participated in this study. Inclusionary criteria included males and females, at least 18 years old, currently employed as a full-time firefighter, and without a known peanut allergy. All participants were employed by the same fire department. This helped ensure a certain amount of consistency in the occupational service that firefighters engaged in throughout the data collection period (i.e., the number and timing of fire calls). Two participants did not wear the sleep monitor as directed, and therefore, their data were removed from the analysis. Participant characteristics of the remaining 38 participants are provided in Table 1.

### 2.3. Protocol

Prior to the first baseline data collection visit, participants were asked to fast without exercising for 8 h and have no caffeine or nicotine for 12 h. During the first visit, participants signed the informed consent document. Age and height were reported by the participant and weight was measured via a stand-on scale (Tanita DC-430U Dual Frequency Total Body Composition Analyzer, Arlington Heights, IL, USA). An actigraphy wristwatch (ActiGraph wGT3X-BT, Pensacola, FL, USA) was given to participants to wear for the first baseline week to monitor sleep. Participants were also asked to fill out visual analog scales (VAS) online via Qualtrics in the morning and evening. 

After the baseline week, participants continued for 7 weeks either in the PB group or the control group. Both groups were asked to stop eating 2 h prior to bedtime and to continue life as they normally would for the 7-week period. Specifically, they were asked to maintain their normal diet, including medications and supplements, and not change their physical activity levels and/or begin a weight loss program throughout their time in the study. In addition, the PB group was asked to consume a 32 g packet of peanut butter 2 h prior to bedtime any five nights of each week, which totaled 35 days of peanut butter consumption out of the 49-day intervention period. Neither group was restricted from peanut butter consumption outside of the 2 h fast prior to bedtime, as they were asked to maintain their normal diet. All participants were asked to continue wearing the actigraphy wristwatch and filling out the bi-daily VAS throughout the 7-week period. 

#### 2.3.1. Actigraphy

The actigraphy wristwatch, a valid measure of activity and sleep, was worn by participants to monitor their sleep throughout the study [29]. It does not show real-time data; thus, it does not provide feedback to promote changes in sleep. The sleep data included sleep latency (i.e., how long it takes to fall asleep), total sleep time, time in bed, sleep efficiency (i.e., time spent asleep/time in bed), wake after sleep onset, number of awakenings per night, and time spent awake per night. It was water-resistant and therefore, appropriate for most of the tasks associated with firefighting.

#### 2.3.2. VAS

The VAS has been shown to be valid and reliable; it included three scales of 0–10 assessing mood, focus, and alertness [30]. The scale for mood ranged from 0 “I feel very down” to 10 “I feel wonderful”, the scale for focus ranged from 0 “I feel very distracted” to 10 “I feel very focused”, and the scale for alertness ranged from 0 “non-coherent” to 10 “I feel very aware of my surroundings”. An investigator texted the survey link to participants at 9:00 AM and 7:00 PM every day throughout the study.

#### 2.3.3. Peanut Butter

Peanut butter was provided to the participants in the PB group. Each serving of peanut butter (Smooth Operator, Peanut Butter & Co, New York, NY, USA) was individually packaged and consisted of 32 g, which is approximately two tablespoons of peanut butter. Each packet contained 190 calories, 15 g of total fat, 8 g of dietary carbohydrates, 2 g of added sugar, and 7 g of protein. Ingredients included peanuts, cane sugar, palm oil, and salt. Participants were instructed to knead the peanut butter packet for 30 s prior to consuming due to the natural separation of the oil. Each participant received a total of 35 packets to take 5 days per week for 7 weeks. 

### 2.4. Statistical Analysis

All analyses were completed with SPSS, version 29 (IBM, Armonk, NY, USA) using an alpha level of 0.05. Data in each group at each time point were screened for outliers (z-score > 3.29 standard deviation units from the group mean) and for the assumption of normality using the Shapiro–Wilk test (α = 0.05). Linear mixed-effect model analysis is robust to violations of normality; therefore, no transformation of the data was made to account for any such reported violations [31]. The intended statistical approach was to complete a series of mixed ANOVAs for the sleep variables and the visual analog scales (VAS) variables. Missing data points from many of the participants, and necessary pairwise deletion, would have resulted in a severely underpowered set of analyses. A linear mixed-effects model approach was selected because this type of analysis can be used to analyze data sets that include partial data for some participants (i.e., when data points are missing) [32]. Changes in the sleep variables latency, sleep efficiency, time in bed, time asleep, wake after sleep onset, number of awakenings, and time spent awake were examined via separate linear mixed-effects model analyses. Individual participants were specified as a correlated random effect to limit the pseudoreplication of results [33]. Averages for each week were computed and designated as the repeated-measures variable week with 8 points (baseline and weeks 2–8). The fixed factors were week and group (PB or control). 

The VAS variables mood, focus, and alertness were similarly analyzed using separate linear mixed-effects models, with individual participants specified as a correlated random effect. Averages for each week in the morning (AM) and evening (PM) were computed and designated as the repeated-measures variable week, with 8 points (baseline and weeks 2–8), and time of day (TOD), with two variables (AM and PM). The fixed factors were week, TOD, and group (PB or control). 

A compound symmetry covariance structure was selected for the repeated-measures factors in all mixed effects models. Tests of fixed effects were generated along with Bonferroni-adjusted post hoc tests, when significant interaction effects were detected, to maintain an alpha level of 0.05. The effect size of the group is reported as Cohen’s *d* and interpreted according to the following recommended benchmarks: small (*d* = 0.20), medium (*d* = 0.50), and large (*d* = 0.80) [34].

## 3. Results

### 3.1. Sleep Variables

Complete descriptive statistics and results from inferential statistical analyses are presented in Table 2 and Table 3, respectively. No outliers in any group of data were detected. There were several violations of the assumption of normality for sleep variables in both groups at different time points. In the PB group, the following violations were observed: latency at weeks 3, 4, and 7; time in bed at weeks 4 and 6; and time asleep at weeks 4 and 6. In the control group, the following violations were observed: latency at all time points; efficiency at weeks 1 and 3; wake after sleep onset at weeks 1, 3, 5, and 6; and time spent awake at weeks 1, 2, 3, 4, 5, 7, and 8. There were no significant interaction effects between group and week for any of the sleep variables latency, efficiency, time in bed, time asleep, wake after sleep onset, number of awakenings, or time spent awake. 

**Figure 1:**
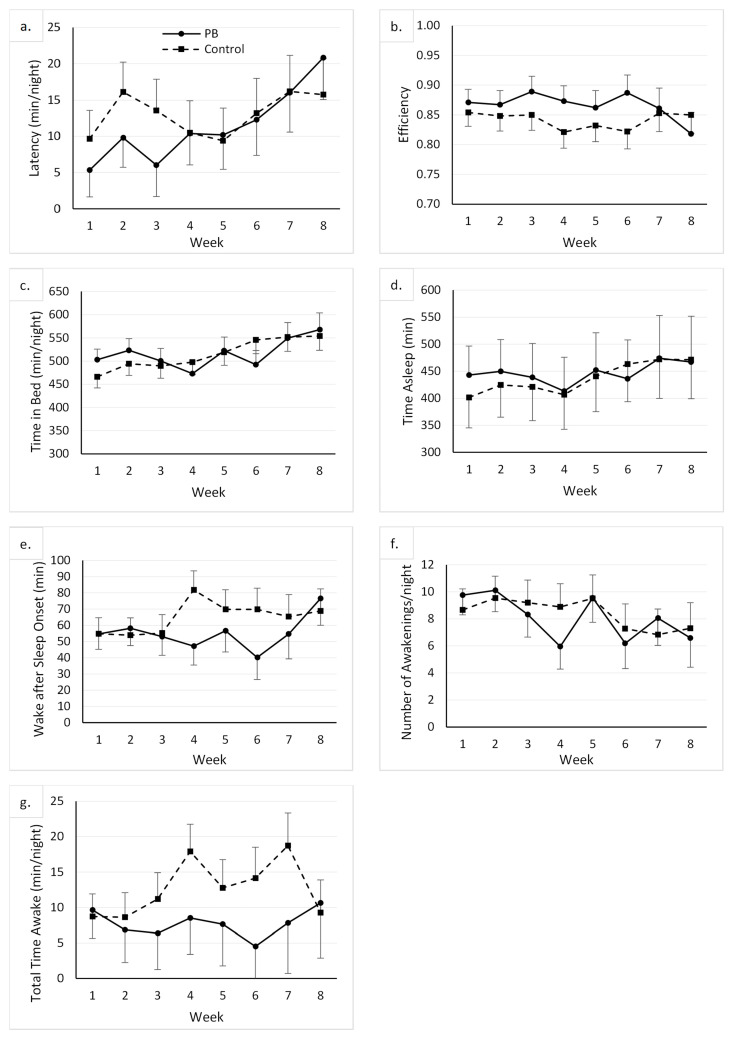
Graphs displaying the descriptive results and changes in sleep variables throughout the course of the study. The weekly average for each participant at each week (time point) was used to compute the mean value for their respective groups [control and peanut butter (PB)], represented as markers on each graph. Whiskers represent standard error of respective mean group values. (**a**): Latency (minutes/night) is a measure of the time before sleep onset; (**b**): Efficiency is a ratio of the time asleep to the time spent in bed; (**c**): Time in Bed (minutes/night) is the time spent in bed each night; (**d**): Time Asleep (minutes/night) is the time spent asleep each night; (**e**): Wake after Sleep Onset (minutes) is the time until the first awakening after sleep onset; (**f**): Number of Awakenings/night is the number of times woken up during the time in bed period; (**g**): Total Time Awake (minutes/night) is the amount of time spent awake during the sleep period.

There was no main effect of the group on any of the sleep variables. There was a main effect of the week on time in bed, which was greater at week 8 than at baseline (mean difference = 76.43 min/night, CI 95% = 4.11, 148.75). There was a main effect of the week on the number of awakenings. However, following the Bonferroni adjustment, none of the post hoc comparisons between time points was significant. The main effects of week on all remaining variables were not significant, as displayed in Figure 1.

### 3.2. VAS Variables

Complete descriptive and inferential statistics for VAS variables mood, focus, and alertness are displayed in Table 4 and Table 5, respectively. No outliers in any group of data were detected. There were violations of the assumption of normality only for the VAS variable mood. In the PB group, the following violations were observed: mood at week 5 in the morning and mood at week 1 in the evening. In the control group, one violation of normality was observed in mood at week 5 in the evening. 

There were no interaction effects between any of the fixed factors group, TOD, or week on mood, focus, or alertness. There was a main effect of TOD on focus, which was higher in AM than in PM (mean difference = 0.20, CI_95_ = 0.07, 0.32). There was also a main effect of week on focus, which was higher in week 6 than at baseline (mean difference = 0.42, CI_95_ = 0.01, 0.82). There was a main effect of TOD on alertness, which was higher in AM than in PM (mean difference = 0.15, CI_95_ = 0.04, 0.26). There were no other significant main effects of group, TOD, or week on mood, focus, or alertness.

## 4. Discussion

The purpose of this study was to determine if 7 weeks of peanut butter consumption prior to bedtime improves sleep quality and/or quantity, as well as subjective feelings of mood, focus, and alertness, in structural firefighters located in the southeastern part of the United States. We hypothesized that there would be an improvement in sleep due to the MUFA content of peanut butter. However, measures of sleep including latency, efficiency, time in bed, time asleep, wake after sleep onset, number of awakenings, and time spent awake did not differ between the PB and control groups at any point throughout the study. In addition, morning and evening VAS variables, including mood, focus, and alertness, did not differ between groups. The lack of improvement in sleep measures may be due to the ineffectiveness of peanut butter as a sleep aid; however, it may also result from a lack of disturbed sleep patterns in this specific firefighter sample. 

Firefighters are on-call for a complete 24 h every 3 days of the week, with loud alarms and announcements broadcasting throughout that time to initiate a quick reactionary response to an emergency. Whether or not an emergency call actually occurs during a shift, the expectancy of a call is enough of a stressor to disturb cardiac autonomic activity [35]. Most firefighters are scheduled shift work with 24 h on call and 48 h off; however, some work regular 8 h day shifts. Mehrdad et al. used the subjective Pittsburgh Sleep Quality Index, a common method to analyze sleep patterns, in over 400 firefighters and compared sleep variables between shift workers and non-shift workers. While there were no differences between groups in sleep efficiency or subjective sleep quality, shift workers demonstrated significantly increased sleep latency times and reduced sleep duration. They also trended toward significance for having more sleep disturbances as well [7]. 

Cary et al. demonstrated that 36% of their firefighter sample had disturbed sleep patterns according to objective sleep measures with actigraphy over a 72 h period. They defined disturbed sleep patterns as having at least two of the following sleep issues: a sleep duration of less than 6 h, a sleep efficiency of less than 85%, a sleep latency of greater than 30 min, and/or a wake after sleep onset of greater than 30 min. Their firefighters averaged sleep latency and wake after sleep onset periods of greater than 30 min, while average sleep duration and efficiency were normal, according to their definition of disturbed sleep [11]. However, the firefighters in our study did not demonstrate disturbed sleep patterns, as they only met one of the criteria mentioned for disrupted sleep, which was an average wake after sleep onset of greater than 30 min. This may be due to the specific shift schedule utilized by the fire department in this study. They work a 24 h on/48 h off shift schedule, which has been shown to allow for a normal sleep pattern to return over the two-day off period [14]. Some fire departments employ a 48 h on/96 h off shift schedule, which still may allow for sleep patterns to return to normal over the four-day off period [14]. Billings et al. demonstrated that the Kelly shift schedule, which consists of 24 h on/24 h off/24 h on/24 h off/24 h on/96 h off, is the most harmful to sleep, as one day is not enough time for sleep patterns to normalize [14]. More research is needed on the effects of shift schedules on sleep impairments in firefighters.

Maintaining a high level of both physical and cognitive performance is essential to properly and safely conduct firefighter duties during emergency situations. Because sleep is important to physical and cognitive function, fire departments and researchers have implemented and evaluated various sleep interventions in hopes of improving sleep quality in these workers. Moreover, nutrition strategies are commonly utilized in health interventions, including sleep health. For instance, some research has demonstrated that consuming carbohydrates prior to bed may lead to shorter sleep latencies, while diets high in protein may improve sleep quality with fewer wake episodes [15,36]. In a review of nutrition studies on sleep health in athletes, it was found that the effect of carbohydrates on sleep health was inconclusive; however, specific foods were shown to improve sleep quantity and/or quality, including tart cherry juice, herbal supplements, kiwifruit, and some protein supplements [37]. 

Peanuts in the form of peanut butter were chosen as the intervention for this study for multiple reasons. Peanut butter is affordable, portable, and pleasant tasting; thus, it is an applicable option for this population and is likely to result in high adherence rates in an intervention [20]. Also, peanuts are high in fat, with most of the fat content being monounsaturated fats (MUFA). MUFA consumption has been correlated to improved sleep health [17]. Although some studies have found total fat intake to be negatively associated with total sleep time, the type of fat in those studies was not characterized. The effect that fat has on sleep depends on the type of fat [36,38]. Sartorius et al. demonstrated improved sleep with MUFA-enriched diets and reduced sleep with saturated fatty acid (SFA)-enriched diets in both humans and mice. Specifically, consumption of an SFA-enriched diet decreased wakefulness during the active part of the day and increased non-rapid eye movement (NREM) sleep, while a MUFA-enriched diet increased rapid eye movement (REM) sleep [39]. This demonstrates an improved quality of sleep in the MUFA group, with resulting increases in wakefulness during the active part of the day compared to the SFA group. Animal studies examining the effects of peanut or MUFA consumption on sleep are limited and should be further investigated.

A higher MUFA to SFA ratio has been shown to be related to better sleep efficiency; a one-point increase in the MUFA to SFA ratio correlated to a 3.11 increased average of sleep efficiency in women, with greater consumption of legumes predicting better sleep efficiency [18,19]. MUFA consumption has also shown sleep benefits in pregnant women. McDonald et al. demonstrated fewer sleeping difficulties in pregnant women with higher MUFA intake. Similarly, in a large sample size, Bennett et al. demonstrated better average sleep durations with no adverse sleep-related symptoms in pregnant women with a higher percentage of MUFA in their diet [40]. Furthermore, Marques-Vidal et al. assessed the dietary intake of macro- and micronutrients and objective measures of sleep in a large sample size of men and women and found that those who slept less than 7 h per day also consumed lower amounts of MUFA [16].

In addition to the high MUFA content, peanuts are also a great source of vitamin E. A serving of peanuts provides over 10% of the recommended dietary allowance of vitamin E, with a content of approximately 20.21 mg per 100 g peanuts [20]. Vitamin E has been shown to improve sleep quality; this may be due to vitamin E’s high antioxidant activity protecting against oxidative stress that may impair sleep [41]. Thongchumnum et al. conducted a randomized controlled trial that assessed vitamin E as an alternative treatment to sedative drugs in individuals with chronic insomnia disorder and found that after one month of daily vitamin E supplementation, the participants had improved sleep quality and a significant reduction in sedative drug usage [42]. Moreover, Alzoubi et al. demonstrated that vitamin E prevented sleep-induced memory impairment in animals, possibly due to its antioxidant activity providing neuroprotection to the hippocampus [1]. Our study used peanut butter as the form of peanut intake. Blanching and mashing the peanuts into peanut butter does not lower the vitamin content of peanuts; however, roasting peanuts may lower the vitamin E content due to its heat sensitivity [20,43]. 

In addition to nutrition interventions, many fire departments and researchers have tried to implement education programs to allow firefighters to gain an understanding of the importance of sleep and advice for improved sleep. For instance, Sullivan et al. implemented the Sleep Health Program, which provided sleep health education, as well as screened for sleep disorders via a questionnaire in over 1000 firefighters. Those who scored positive for sleep disorders on the questionnaire were referred to a sleep clinic for further help. They found no differences in self-reported sleep from the educational intervention but did find a reduced number of injuries and disability-related work loss which may be related to sleep quality [10]. 

A systematic review of behavioral and cognitive sleep health interventions in the general population without a clinical sleep disorder demonstrated that the only two areas of improved sleep health were subjective sleep quality and sleep duration [44]. Some of these interventions require a trained facilitator. Jang et al. implemented and evaluated a sleep intervention in 39 Korean firefighters called Firefighter’s Therapy for Insomnia and Nightmares (FIT-IN). This intervention consisted of three therapist-led sessions that focused on sleep education, nightmare education, and relaxation therapy, and the last session was to help reinforce positive changes in sleep habits. The firefighters completed a sleep diary for two weeks, which was assessed to reveal significant improvements in sleep efficiency, sleep onset latency, number of awakenings, and time in bed resulting from the intervention. Also, subjective questionnaires demonstrated reduced occurrences of insomnia and nightmares [45].

Sleep disturbances in firefighters may also vary according to the number of calls received and emergency situations encountered. We measured sleep with actigraphy over an 8-week period, and on average, the firefighters did not demonstrate disturbed sleep patterns in terms of total time in bed, sleep latency, and sleep efficiency. One interesting point to note is the regular occurrence of multiple periods of sleep in a 24 h period, which was summed to provide total time in bed. For instance, a firefighter may sleep 4 h during the night and then another 2 h during the day for a total of 6 h of sleep for the 24 h period. Although the end product of total time in bed and time asleep may seem like the firefighters do not have disturbed sleep patterns, a closer look may reveal the varied sleep that each firefighter may have on different days of the week. Napping has been shown to benefit both the physical and cognitive performances of athletes who may have had sleep loss overnight [36]. For instance, Waterhouse et al. demonstrated that a 30 min nap in athletes who only slept 4 h at night improved running sprint performances and feelings of alertness and sleepiness [46]. Therefore, the acute effects on physical and cognitive performance, in addition to the chronic effects on health from split sleep periods, should be further investigated in firefighters.

In addition to objective actigraphy and morning/night energy questionnaires, asking our participants to keep a sleep diary would have improved our study, as it would have subjectively validated the objective sleep measures of the actigraphy. Conversely, many sleep studies primarily utilize subjective questionnaires, such as the Pittsburgh Sleep Quality Index, to determine sleep measures. However, data are needed from both subjective sleep diaries and/or questionnaires and objective sleep monitors to better determine when the participant is awake and asleep [25]. A second limitation of the study is the lack of current diet characterization and daily dietary logs. Participants were asked to maintain their normal diet to determine if the addition of consuming peanut butter prior to bed altered sleep. Dietary logs would have provided confirmation that participants did maintain their normal diet throughout the 8-week period. However, dietary records, diet history, and recalls can be difficult for individuals to fill out accurately and often lead to missing information being recorded [47,48,49]. Limitations for recall include dependence on memory and knowing all the ingredients to record [47,50]. A firefighter’s lack of sleep may contribute to an inability to fill logs out accurately and within the firehouse; a firefighter does not regularly make their own meals, which could lead to missed ingredients. Furthermore, in Joe et al.’s review of diets among firefighters, only three out of seventeen studies that focused on dietary interventions for firefighters required participants to maintain food diaries [51]. One may consider the use of an application (App) for tracking, but studies have shown that they do not provide accurate calculations either [52]. 

In addition to potential inaccuracies, participants self-reporting dietary intake information may ultimately influence the dietary behaviors being monitored, as described by the Hawthorne effect and reported in past nutrition intervention reports [53,54,55]. By not controlling or formally monitoring dietary intake or consumption of substances that could alter sleep (caffeine, alcohol, medications, etc.), we hoped to preserve the generalizability of the study results. Moreover, pre-existing health conditions, except for a peanut allergy, that may affect sleep were not part of the exclusionary criteria. Those taking medications for health conditions were asked to maintain their medication regimen throughout the study. 

Finally, the findings are limited to a certain extent by the loss of some data from participants not wearing the sleep monitor as instructed, despite reminders and regular in-person visits from study team members. Data were analyzed in a way to preserve as much as possible to address the research question, yet this loss of some data should be considered when interpreting the results.

One of the key strengths of this study is that it was a field intervention in which participants were tasked with implementing the instructions on their own under circumstances that mirrored their everyday occupational demands and schedules. This aspect of the methodology contributed to the ecological validity of the study, making the results of the intervention more generalizable to the population of interest (shift-working firefighters). Considerations from this report can be borne in mind when attempting to develop nutritional interventions for improved sleep.

Future explorations should assess peanut consumption on sleep quality, using both subjective and objective sleep measurements, as well as daily cognitive performance in firefighters. Explorations should also determine an accurate and appropriate dietary assessment for the firefighter population to aid in the confirmation that sleep effects were associated with the addition of peanut butter and not another dietary change.

## 5. Conclusions

Peanut butter did not positively or negatively impact objective measures of sleep as measured by actigraphy or subjective feelings of energy, mood, and alertness, as measured by visual analog scales, over a 7-week period in full-time firefighters. However, this study design may be more effective and robust in populations that display greater sleep impairments. Thus, further research is needed to determine the effectiveness of peanut butter on sleep quantity and/or quality in firefighters.

## Figures and Tables

**Table 1:** Demographic and anthropomorphic information for participants in the control group and the peanut butter (PB) group. Age and anthropomorphic data are presented as mean values followed by standard deviation values in parentheses.

	Control (*n* = 18; 0 Women)	PB (*n* = 20; 1 Woman)	All (*n* = 38; 1 Woman)
Age (years)	37.06 (6.45)	33.35 (9.07)	35.11 (8.05)
Height (cm)	179.07 (7.19)	178.31 (7.49)	178.67 (7.26)
Weight (kg)	100.74 (26.97)	101.13 (26.25)	100.95 (26.23)
BMI (kg/m^2^)	31.15 (7.72)	31.89 (8.36)	31.55 (7.97)
Body Fat (%)	24.26 (7.64)	25.96 (10.13)	25.18 (9.00)
Race (*n*)			
White	17	16	33
Black or African American	1	3	4
Asian	0	1	1
Ethnicity (*n*)			
Hispanic	1	1	2
Non-Hispanic	17	19	36

**Table 2:** Descriptive statistics for sleep variables for each week of the intervention period for the control and peanut butter (PB) groups. The mean value is presented followed by the standard deviation in parentheses. Effect size between groups at each week is presented as Cohen’s *d*.

	Week	Control	PB	Effect Size
Latency	1	9.66 (16.59)	5.35 (16.59)	0.26
	2	16.10 (17.52)	9.80 (18.18)	0.40
	3	13.54 (18.31)	6.03 (19.40)	0.49
	4	10.49 (18.74)	10.39 (19.38)	0.01
	5	9.38 (19.21)	10.18 (21.23)	0.05
	6	13.19 (20.34)	12.29 (22.04)	0.06
	7	16.19 (21.10)	16.02 (24.28)	0.01
	8	15.75 (21.10)	20.85 (25.91)	0.38
Efficiency	1	0.85 (0.10)	0.87 (0.10)	0.17
	2	0.85 (0.11)	0.87 (0.11)	0.20
	3	0.85 (0.11)	0.89 (0.12)	0.42
	4	0.82 (0.11)	0.87 (0.12)	0.57
	5	0.83 (0.11)	0.86 (0.13)	0.34
	6	0.82 (0.12)	0.89 (0.13)	0.75
	7	0.85 (0.13)	0.86 (0.15)	0.09
	8	0.85 (0.13)	0.82 (0.16)	0.37
Time in Bed	1	465.97 (102.04)	503.09 (102.04)	0.36
	2	494.38 (108.02)	523.17 (112.24)	0.29
	3	489.80 (113.06)	500.51 (120.02)	0.11
	4	497.84 (115.80)	473.03 (119.91)	0.26
	5	519.01 (118.34)	522.48 (131.75)	0.04
	6	545.77 (126.13)	492.34 (136.96)	0.61
	7	551.76 (130.96)	549.39 (151.26)	0.03
	8	554.15 (130.96)	567.77 (161.64)	0.16
Time asleep	1	401.53 (238.75)	443.03 (238.75)	0.17
	2	424.59 (252.76)	449.80 (262.64)	0.75
	3	420.88 (264.56)	438.70 (280.88)	0.32
	4	406.47 (270.99)	412.98 (280.63)	0.26
	5	440.64 (278.10)	452.08 (308.34)	0.31
	6	462.99 (295.19)	436.06 (320.57)	0.13
	7	471.85 (306.51)	473.76 (354.06)	0.28
	8	471.18 (306.51)	466.97 (378.38)	0.25
Wake after Sleep Onset	1	54.79 (42.14)	54.66 (42.14)	0.00
	2	53.92 (45.35)	58.14 (47.50)	0.10
	3	55.24 (48.06)	53.05 (51.78)	0.05
	4	81.81 (49.58)	47.10 (51.74)	0.86
	5	69.85 (51.26)	56.59 (58.15)	0.34
	6	69.83 (55.30)	40.23 (60.97)	0.76
	7	65.29 (57.94)	54.69 (68.62)	0.28
	8	68.79 (57.94)	76.56 (74.12)	0.21
Number of awakenings	1	8.67 (6.55)	9.76 (6.55)	0.17
	2	9.54 (6.86)	10.12 (7.08)	0.09
	3	9.20 (7.12)	8.33 (7.48)	0.15
	4	8.89 (7.26)	5.96 (7.47)	0.50
	5	9.51 (7.41)	9.56 (8.08)	0.01
	6	7.27 (7.78)	6.19 (8.35)	0.20
	7	6.84 (8.03)	8.07 (9.09)	0.24
	8	7.31 (8.03	6.59 (9.64)	0.14
Time spent awake	1	8.72 (13.53)	9.64 (17.88)	0.05
	2	8.62 (14.72)	6.86 (20.56)	0.10
	3	11.19 (15.74)	6.37 (22.84)	0.27
	4	17.90 (16.32)	8.52 (22.83)	1.08
	5	12.75 (16.97)	7.65 (26.21)	0.28
	6	14.13 (18.55)	4.54 (27.71)	0.53
	7	18.72 (19.57)	7.82 (31.77)	0.60
	8	9.27 (19.57)	10.63 (34.66)	0.11

**Table 3:** Inferential statistics for sleep variables. Outputs of linear mixed-effects model analyses were used to generate estimates of fixed effects for the factors group [control or peanut butter (PB)] and week (baseline and weeks 2–8).

Interaction and Main Effects		*df*	*F*	*p*
Latency	Group	1, 34.69	0.12	0.726
	Week	7, 130.38	1.98	0.063
	Group × Week	7, 130.83	0.69	0.678
Efficiency	Group	1, 38.95	0.84	0.364
	Week	7, 135.83	0.45	0.870
	Group × Week	7, 135.83	0.71	0.662
Time in Bed	Group	1, 37.37	0.00	0.955
	Week	7, 133.22	2.88	0.008 *
	Group × Week	7, 133.22	1.02	0.417
Time asleep	Group	1, 37.43	0.07	0.796
	Week	7, 132.43	1.72	0.110
	Group × Week	7, 132.43	0.53	0.808
Wake after Sleep Onset	Group	1, 40.79	0.81	0.374
	Week	7, 139.42	0.66	0.706
	Group × Week	7, 139.42	1.18	0.320
Number of awakenings	Group	1, 36.84	0.03	0.865
	Week	7, 131.49	2.11	0.047 *
	Group × Week	7, 131.49	0.78	0.605
Time spent awake	Group	1, 35.12	2.26	0.142
	Week	7, 140.68	0.65	0.711
	Group × Week	7, 140.68	0.76	0.619

* = significant effect.

**Table 4:** Descriptive statistics for visual analog scale (VAS) variables mood, focus, and alertness for each week of the intervention period for the control and peanut butter (PB) groups, in both the morning (AM) and evening (PM). The mean value is presented followed by the standard deviation in parentheses. Effect size between groups at each time of day is presented as Cohen’s *d*.

Variable	Week	Control		PB		Effect Size	
		AM	PM	AM	PM	AM	PM
Mood	1	7.22 (0.28)	7.50 (0.28)	6.97 (0.28)	7.28 (0.28)	0.20	0.18
	2	7.19 (0.28)	7.37 (0.28)	7.14 (0.28)	7.13 (0.28)	0.04	0.20
	3	7.21 (0.28)	7.32 (0.28)	7.23 (0.28)	7.42 (0.28)	0.02	0.08
	4	7.10 (0.28)	7.03 (0.28)	7.00 (0.28)	7.19 (0.28)	0.07	0.13
	5	7.32 (0.29)	7.22 (0.28)	7.28 (0.28)	7.30 (0.28)	0.03	0.06
	6	7.10 (0.29)	7.12 (0.29)	7.22 (0.28)	7.35 (0.28)	0.10	0.19
	7	7.31 (0.29)	7.24 (0.29)	7.25 (0.28)	7.34 (0.28)	0.06	0.08
	8	7.34 (0.30)	7.03 (0.29)	7.27 (0.29)	7.21 (0.29)	0.06	0.16
Focus	1	7.38 (0.34)	7.04 (0.34)	6.62 (0.33)	6.65 (0.33)	0.51	0.27
	2	7.38 (0.34)	7.13 (0.34)	7.17 (0.33)	6.89 (0.33)	0.14	0.16
	3	7.45 (0.34)	7.17 (0.34)	7.17 (0.33)	7.17 (0.33)	0.19	0.00
	4	7.24 (0.34)	7.04 (0.34)	7.15 (0.33)	7.11 (0.33)	0.06	0.05
	5	7.39 (0.34)	7.35 (0.34)	7.26 (0.33)	6.71 (0.33)	0.09	0.45
	6	7.42 (0.34)	7.26 (0.34)	7.46 (0.33)	7.21 (0.33)	0.03	0.03
	7	7.56 (0.35)	7.06 (0.35)	7.41 (0.33)	7.22 (0.33)	0.11	0.12
	8	7.45 (0.35)	7.20 (0.35)	7.19 (0.34)	7.34 (0.34)	0.19	0.10
Alertness	1	8.15 (0.35)	7.82 (0.35)	7.13 (0.34)	7.32 (0.34)	0.67	0.33
	2	8.12 (0.35)	7.93 (0.35)	7.48 (0.34)	7.16 (0.34)	0.42	0.51
	3	8.03 (0.35)	7.97 (0.35)	7.49 (0.34)	7.63 (0.34)	0.36	0.23
	4	7.89 (0.35)	8.03 (0.35)	7.54 (0.34)	7.63 (0.34)	0.24	0.27
	5	8.19 (0.35)	7.99 (0.35)	7.66 (0.34)	7.25 (0.34)	0.36	0.50
	6	8.07 (0.35)	7.70 (0.35)	7.74 (0.34)	7.62 (0.34)	0.22	0.05
	7	7.95 (0.36)	7.78 (0.36)	7.71 (0.34)	7.40 (0.34)	0.17	0.26
	8	8.28 (0.36)	7.89 (0.36)	7.63 (0.35)	7.50 (0.35)	0.46	0.27

**Table 5:** Inferential statistics for visual analog scale (VAS) variables mood, focus, and alertness. Output of linear mixed-effects model analyses were used to generate estimates of fixed effects for the factors group [control or peanut butter (PB)], week (baseline and weeks 2–8), and time of day [TOD; morning (AM) and evening (PM)].

**Interaction Effects**		***df***	***F***	***p***
Mood	Week × Group	7, 518.43	0.61	0.752
	TOD × Group	1, 518.05	0.69	0.405
	Week × TOD × Group	7, 518.01	0.19	0.987
Focus	Week × Group	7, 518.26	1.34	0.229
	TOD × Group	1, 517.95	0.72	0.396
	Week × TOD × Group	7, 517.92	0.72	0.656
Alertness	Week × Group	7, 518.15	1.48	0.170
	TOD × Group	1, 517.93	0.62	0.432
	Week × TOD × Group	7, 517.91	0.64	0.721
**Main effects**		***df***	***F***	***p***
Mood	Week	7, 518.43	0.73	0.645
	TOD	1, 518.05	0.91	0.342
	Group	1, 36.93	0.00	0.992
Focus	Week	7, 518.26	2.20	0.033 *
	TOD	1, 517.95	9.29	0.002 *
	Group	1, 36.81	0.19	0.668
Alertness	Week	7, 518.15	0.80	0.592
	TOD	1, 517.93	6.97	0.009 *
	Group	1, 36.86	1.28	0.266

* = significant effect.

## Data Availability

Documents with statistical analysis output are available at the following link to the Open Science Framework repository: https://osf.io/j3bx4/ (accessed on 11 March 2024).
